# Phase separation and viral factories: unveiling the physical processes supporting RNA packaging in dsRNA viruses

**DOI:** 10.1042/BST20231304

**Published:** 2024-09-26

**Authors:** Cyril J. Haller, Julia Acker, A. Emilia Arguello, Alexander Borodavka

**Affiliations:** 1 Department of Chemical Engineering and Biotechnology, University of Cambridge, Cambridge, U.K.; 2 Department of Biochemistry, University of Cambridge, Cambridge, U.K.

**Keywords:** RNA structure, RNA:RNA interactions, RNP granules, viral factories, X-ray RNA footprinting

## Abstract

Understanding of the physicochemical properties and functions of biomolecular condensates has rapidly advanced over the past decade. More recently, many RNA viruses have been shown to form cytoplasmic replication factories, or viroplasms, via phase separation of their components, akin to numerous cellular membraneless organelles. Notably, diverse viruses from the *Reoviridae* family containing 10–12 segmented double-stranded RNA genomes induce the formation of viroplasms in infected cells. Little is known about the inner workings of these membraneless cytoplasmic inclusions and how they may support stoichiometric RNA assembly in viruses with segmented RNA genomes, raising questions about the roles of phase separation in coordinating viral genome packaging. Here, we discuss how the molecular composition of viroplasms determines their properties, highlighting the interplay between RNA structure, RNA remodelling, and condensate self-organisation. Advancements in RNA structural probing and theoretical modelling of condensates can reveal the mechanisms through which these ribonucleoprotein complexes support the selective enrichment and stoichiometric assembly of distinct viral RNAs.

## Introduction

The spatiotemporal organisation of biomolecules within crowded cellular milieux is essential for maintaining cellular functions [1]. Membrane-bound sub-cellular compartments such as the endoplasmic reticulum or mitochondria offer a physical barrier for different cellular components and processes, while membrane-less compartments do not rely on a surrounding phospholipid bilayer. Their formation is driven by liquid-liquid phase separation (LLPS), a process that describes the spontaneous de-mixing of biopolymers into a dense phase enriched in biomolecules and a biomolecule-depleted phase. The dense phase often displays liquid-like behaviour as it can fuse, wet surfaces, and reversibly deform. These dynamic properties allow for rapid exchange of biomolecules with the surroundings, which is thought to fine-tune biochemical reactions by either concentrating or sequestering reaction partners. These biomolecular condensates are involved in organisation of various key cellular functions, ranging from cell division to a cellular stress response [2], and include stress granules, P-bodies, and the nucleolus [3–6]. It is estimated that roughly one-fifth of the eukaryotic proteome is partitioned into membraneless organelles (MLOs), a figure comparable to the 40% contained within membrane-bound structures [7]. Despite first being described over a century ago, interest in condensates has developed relatively recently following an improved understanding of the physical processes underlying macromolecular condensation into distinct phases [3].

The number and nature of resident biomolecules can vary greatly across different MLOs. In contrast with large molecular complexes typically characterised by defined molecular stoichiometries and fixed binding sites between interacting partners, neither parameter is fixed within MLOs and can dynamically change in response to the environment. The transient nature and dynamic complexity of these assemblies significantly impede their characterisation.

LLPS is typically driven by weak, multivalent interactions, as the energetic contribution of transient attractive interactions within the condensate (also known as demixing) offsets the entropic cost of mixing [8]. The resulting droplets are enriched in proteins and nucleic acids with a propensity to form such weak multivalent interactions. The constituents of condensates that are essential for condensate formation are generally classified as ‘scaffolds,’ while the term ‘clients’ describes biomolecules that are recruited into condensates due to attractive interactions with scaffolds [9]. Unlike scaffolds, clients are not actively retained within MLOs. Instead, clients exhibit reduced mobility when diffusing across the boundary between phases [10], appearing as dynamic residents of condensates. While proteins and RNAs can independently nucleate LLPS, they may also interact to drive the formation of ribonucleoprotein (RNP) granules in which molecules may sample multiple conformations to interact with diverse binding partners [11,12].

### Viroplasms as models for studying RNA selectivity of biomolecular condensates

Many virus-induced cytoplasmic inclusions also known as viroplasms or viral replication factories, present an attractive model system to study RNP granule formation. Viroplasms have long been identified as viral genome replication and assembly sites. Viroplasm assembly can be induced in a controlled fashion by infecting a cell, and morphological changes can be monitored throughout infection. Importantly, the genetic parsimony of viruses limits the compositional complexity of viroplasms. Exploring the relevance of individual LLPS components, i.e. viral protein scaffolds, in a biologically relevant context can be achieved through genetic manipulations, such as reverse genetics, or using conditional, e.g. temperature-sensitive, mutants. The well-defined biological function of viral condensates, carried out by a minimalistic collection of components, may shed light on the role of phase transitions in viral assembly. Double-stranded (ds)RNA viruses with a segmented genome, such as rotaviruses, provide an excellent model to study MLOs as these viruses form condensates that selectively recruit eleven non-identical RNAs required for genome packaging. Rotaviruses are particularly useful models, as their dsRNA genomes that would trigger a cellular immune response are never exposed outside of assembled particles [13]. Hence, all pre-genomic ssRNAs selected for packaging prior to replication are positive-sense ssRNAs that accumulate in viroplasms.

**Figure 1.**
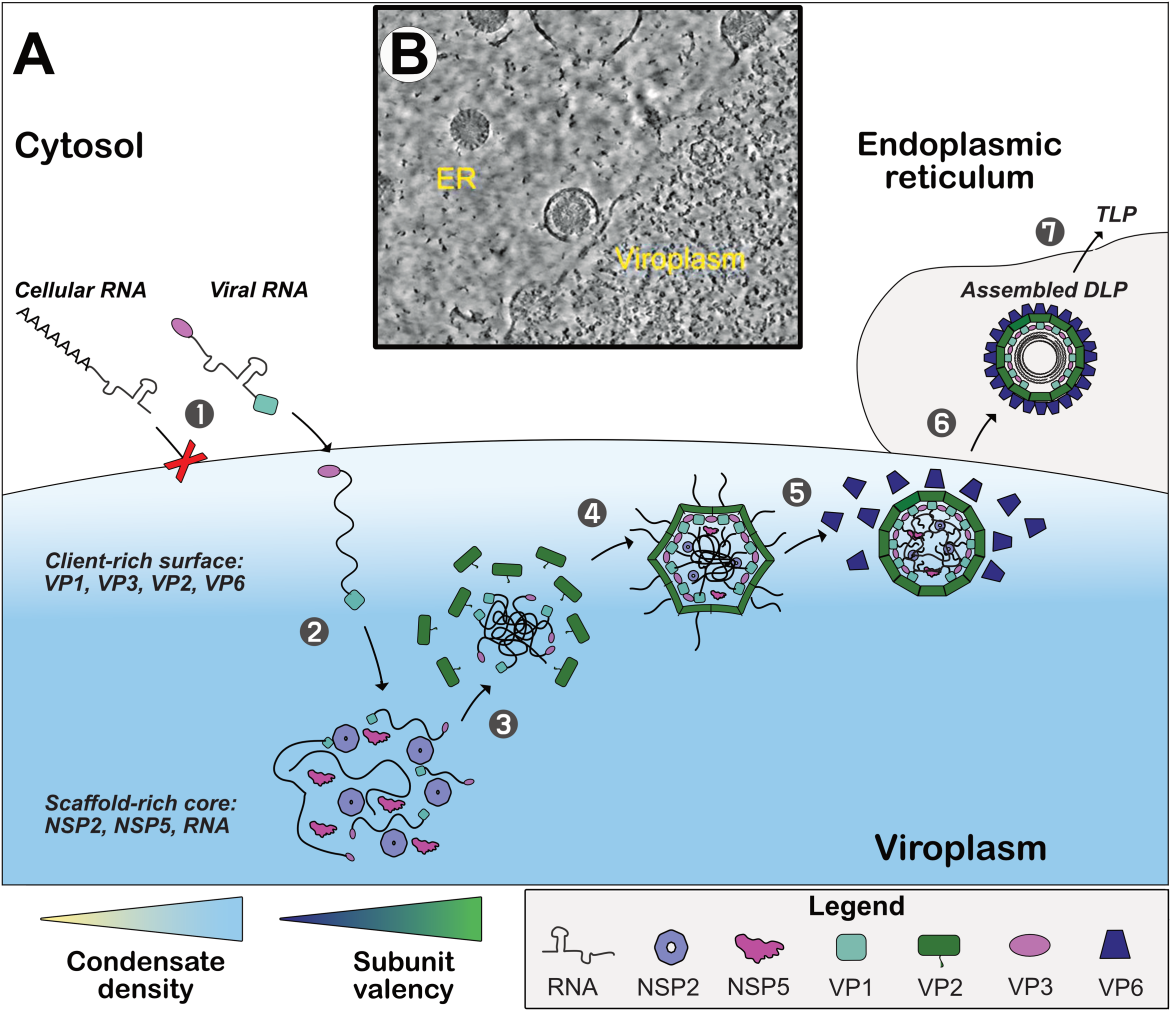
Rotavirus replication takes place in viroplasms formed via phase separation. (**A**) A proposed model of the rotavirus viroplasm and its key contributions to the viral replication cycle. Viral transcription following the uncoating of endocytosed triple-layered particles (TLPs) results in the extrusion of viral mRNAs followed by their translation. This creates a cytoplasmic pool of viral proteins, including viroplasm-forming NSP5 and NSP2. These proteins undergo LLPS to form early viroplasms, which recruit additional clients required for viral assembly. RNA recruitment is selective, favouring viral transcripts over cellular ones, ensuring the enrichment of eleven distinct types of viral RNAs (step 1). RNA relocalisation to the viroplasm is associated with RNA remodelling, promoting interactions with other RNAs and viral proteins (step 2). Viral inner capsid protein VP2 shells assemble around growing clusters of up to eleven distinct single-stranded RNAs in distinct micro-domains (step 3). The remaining unpackaged regions of these RNA complexes fill pre-assembled VP2 shells (step 4). Packaged RNA undergoes replication before or during assembly of double-layered particles (DLPs, step 5). The endoplasmic reticulum (ER)-resident viral protein NSP4 recruits DLPs assembled in viroplasms into the ER (step 6), where TLPs are assembled (step 7). (**B**) Electron microscopy image of rotavirus viroplasm with NSP2 octamers dispersed throughout the viroplasm and particle intermediates visible only at the surface of the condensate [16].

Viroplasm formation selectively increases local concentrations of viral constituents for assembly while minimising the potential for spurious interactions that interfere with the process. For example, the rotavirus non-structural protein NSP5 binds with high affinity to the viral polymerase VP1, recruiting it to viroplasms [14]. Simultaneously, the viral RNA polymerase VP1 binds the conserved 3′ sequence in all viral ssRNAs to promote selective ssRNA enrichment, as evidenced by the accumulation of viral transcripts within viroplasms and the exclusion of cellular transcripts [15]. Sequence-specific VP1 binding limits non-specific interactions with non-viral ssRNAs, highlighting the importance of balancing high-affinity interactions required for selectivity with the weaker ones that maintain fluid-like properties of viroplasms. The selectivity conferred by condensate interfaces, therefore, controls the constituents within phases and the diffusion between them, determining RNA colocalisation and potentially ssRNA remodelling and the formation of transient RNA:RNA contacts (Figure 1A). Cellular components such as lipid droplets, actin filaments and tubulin have also been implicated in rotavirus viroplasm dynamics, though the exact molecular mechanisms through which these contribute to viral replication remain inconclusive [17–19].

Computational modelling predicts that subunit oligomerisation and assembly of viral particles would progressively reduce network contributions required for partitioning until the entropic cost of their condensation is too great, which could theoretically cause the assembled particles with packaged RNA to be radially expelled from viroplasms (Figure 1A) [8]. Maintaining multiple smaller viroplasms rather than fewer large ones provides a greater overall surface area, which could, in theory, allow for more subunit exchange with the cytosol [20,21]. Limiting viroplasm size also decreases their radial distance, reducing the time required for viral components to diffuse within and dynamically interact to assemble nascent particles [20].

### Condensates remodel RNAs and are influenced by their structures

**Figure 2.**
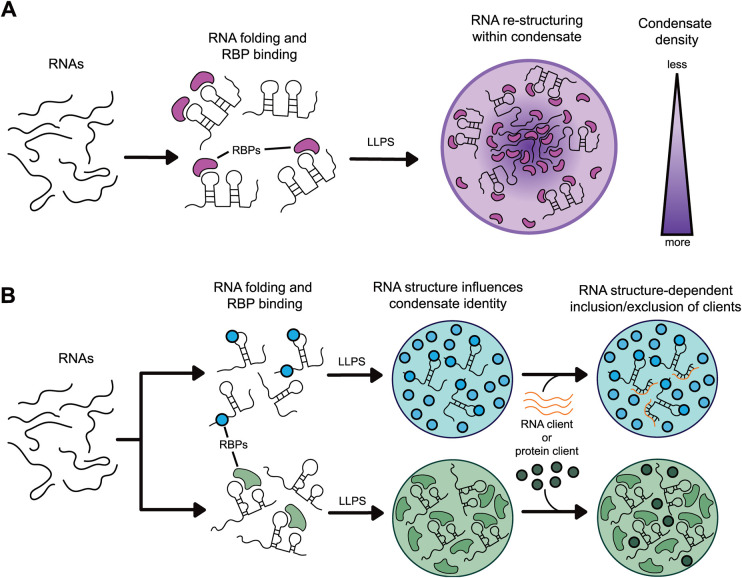
Current understanding of RNA structure in condensates. (**A**) The environment inside RNP condensates can remodel ssRNA structure. Inside condensates, large folded ssRNAs can undergo partial unfolding reforming extensive inter-molecular base-pairing with adjacent ssRNAs. (**B**) A model of RNA enrichment controlled by the RNA structure that may affect condensate's selectivity and composition by presenting distinct binding sites that recruit additional clients (ssRNAs and proteins). Further partitioning of ssRNAs (orange) or RNA-binding proteins (RBP, green) through base-pairing or binding site display, respectively, allow dynamic control of the RNP granules’ composition.

Theoretical models of phase-separated RNA-protein mixtures suggest that the solvent environment inside condensates can denature nucleic acid secondary structure and stabilise single-stranded conformations (Figure 2A) [22]. Coarse-grained simulations of (CAG)*_n_* trinucleotide repeats similarly predicted the melting of hairpin-like structures upon condensate entry [23]. Simple coacervates consisting of tRNAs and cationic peptides allow the RNAs to maintain short-range interactions but not long-range secondary or tertiary contacts [24]. Experimental data suggest that the elevated local concentration of RNA-binding proteins can remodel RNA structures following their selective partitioning into condensates [22,25]. It should be noted that most studies of RNA structures inside condensates have been carried out in simple systems with short RNA sequences due to the challenges of investigating larger transcripts, particularly *in situ* [26]*.* Some of these limitations have been overcome using RNA chemical structure probing and single-molecule imaging [25]. For larger mRNAs, structure probing via 2′-hydroxyl acylation analysed by primer extension and mutational profiling (SHAPE-MaP) approach revealed reorganisation of *CLN3* mRNA upon entrance into Whi3 protein granules [25].

Just as condensate's properties alter RNA structure, growing evidence suggests that RNA structure affects condensates (Figure 2B). Research of RNP granules showed that highly structured RNA regions within condensate-forming transcript *CLN3* prevent base-pairing with non-self RNAs and, thus, promote the formation of discrete condensates with the PolyQ-containing protein Whi3 [25]. More flexible RNAs, such as *BNI1*, allow for RNA-RNA hybridisation with other transcripts, enabling the co-partitioning of mRNA pairs into the same condensate or selective inclusion of a client mRNA into an already-formed droplet. Similarly, the addition of highly structured non-coding RNA NEAT1 stimulated FUS protein droplet formation in environments where phase separation is not typically observed [27].

Conversely, in other systems, RNA secondary structure can favour more fluid states or hinder LLPS altogether. For instance, long AU-rich RNAs with unstructured 3′-UTRs partition into and form a large, mesh-like MLO network of TIS granules [28], while the formation of P-granules in *Caenorhabditis elegans* is deterred by the presence of structured RNAs [29].

Together, these results support a model where RNA structure influences several aspects of condensate formation and dynamics: licensing interactions with distinct protein constituents, mediating or inhibiting the selective uptake of client RNAs into formed granules, or establishing overall condensate morphology. The ability of RNAs to alter the viscosity of RNP granules remains an understudied topic that paves the way for understanding the formation of biphasic condensates with subcompartments differing in RNA and protein composition.

In light of these findings, the structural reorganisation of RNAs may also be a prerequisite for the stoichiometric co-assembly of eleven distinct rotavirus transcripts inside viroplasm condensates [30]. Nascent RNA base-pairing in viral transcripts may be remodelled upon recruitment into viroplasms, where the newly exposed RNA interfaces can re-establish long-range interactions required for RNA assortment (Figure 1A). Interestingly, following the discovery of phase separation as the driving force for viroplasm formation in rotaviruses, other related viruses of the *Reoviridae* family have been found to assemble their replication factories using similar principles. These include 10-segmented mammalian and avian reoviruses and bluetongue virus [31–33]. The assembly pathways of dsRNA viruses in viroplasms remain largely unclear and may vary between viral species. The prominent model based on biochemical and structural studies of rotaviruses describes the co-assembly of capsids around a multi-RNA complex before its replication akin to other (+)-sense ssRNA viruses (Figure 1A) [16,34]. Most recent structural studies of bluetongue virus support this model, whereby infection virions are produced through the co-assembly of viral capsids around the RNA [35]. An improved understanding of RNA behaviour and its interactions within condensates would shed light on segmented RNA virus assembly mechanisms.

### Promising tools to study RNA structure inside condensates

**Figure 3.**
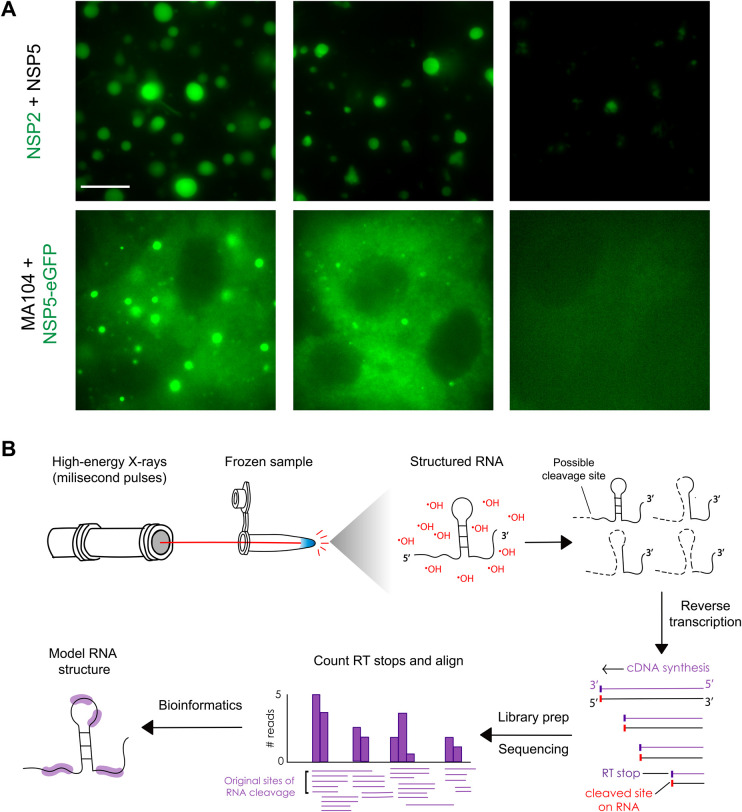
RNA structure probing in condensates. Organic solvents traditionally used for RNA structure probing, such as DMSO, can disrupt liquid-like condensates both *in vitro* and in cellulo. (**A**) Top: *in vitro* condensates formed by mixing of recombinant rotavirus proteins NSP5 and an Atto-488-tagged NSP2 are partially dissolved in the presence of 5% dimethylsulfoxide (DMSO) and fully dissolved after the addition of 10% DMSO as used in SHAPE-MaP. Bottom: Replication factories formed upon infection with RV are fluorescently labelled via partitioning of transiently expressed NSP5-eGFP into viroplasms. Viroplasms disappear in the presence of 5% and 10% DMSO. Scale bar = 10 µm. (**B**) XFP can be used to probe RNA structure inside condensates. Frozen biological or reconstituted samples are exposed to high-energy X-ray radiation, which generates ·OH radicals from water radiolysis. Radical-mediated cleavage of the RNA at solvent-exposed sites is recorded as reverse transcriptase (RT) stops during next-generation sequencing and is used to model RNA structure.

With RNA structure emerging as a defining feature in modulating LLPS, tools for elucidating RNA structure within condensates may reveal new functions of condensates in mediating RNA selection and assembly. Several RNA structure probing techniques, such as dimethyl sulfate sequencing or SHAPE-MaP, rely on the chemical reactivity of RNA functional groups (e.g. nitrogens involved in base-pairing or 2′-OH groups) as a proxy for individual nucleotide flexibility and accessibility, which can then be used to more accurately model RNA secondary structure [36]. SHAPE-MaP has already been deployed in the context of LLPS to understand the contributions of RNA structure to condensate behaviour, including SARS-CoV-2 genome packaging and perinuclear droplet formation in fungi [25,26,37–39]. It is crucial to consider that this kind of chemical probing relies on the extraneous addition of a small molecule, usually dissolved in polar organic solvents such as dimethylsulfoxide (DMSO) or ethanol in concentrations as high as 10% both *in vitro* [25,36–38,40] and *in vivo* [40–43] (Table 1), which can disrupt condensate integrity and compromise the accuracy of results. Caution should be taken when using these organic solvents for RNA structure probing within liquid-like condensates, as these molecules can disrupt LLPS (Figure 3A).

**Table 1.** Experimental conditions of cited RNA structural probing studies

Method	Experiment	Solvent concentration (%)	Solvent used	References
SHAPE	*In vitro*	10	DMSO	[25]
SHAPE	*In vitro*	10	DMSO	[36]
SHAPE	*In vitro*	10	DMSO	[37]
SHAPE	*In vitro*	9.1	DMSO	[38]
SHAPE	*In vivo*	10	DMSO	[41]
SHAPE	*In vivo*	10	DMSO	[42]
SHAPE	*In vivo*	5	DMSO	[43]
DMS	*In vivo*	8	Ethanol	[40]
DMS	*In vitro*	8.4	Ethanol	[40]

In addition to individual limitations, traditional chemical probing methods rely on reporting chemistries (e.g. hydrolysis, methylation, acylation) with timescales often in the range of minutes to several hours that are too long to capture fast, local rearrangements of RNA structure inside condensates. This timing can be even harder to control when probing RNA in living cells, as membrane permeability can vary vastly among structure-probing molecules, and some reagents might even dissolve RNP granules. Thus, these strategies have so far been limited to *in vitro*-reconstituted systems and might fail to recapitulate the properties of RNP granules in their native environment. Hydroxyl radical footprinting (HRF) has emerged as a promising technique to probe RNA structure on faster timescales. HRF works by mapping regions of a biomolecule susceptible to hydrolysis by Fenton chemistry-generated hydroxyl radicals (·OH) in the second timescale. It has been chiefly applied to elucidate protein structure [44] and tertiary RNA structure in kinetic and equilibrium conditions [45,46]. This timescale can be dramatically shortened using high-energy radiation, such as that derived from synchrotrons, lasers, or pulse UV sources, generating ·OH from the radiolysis of water or the photolysis of hydrogen peroxide in a µs to ms timescale [44].

The use of high-flux synchrotron X-rays has been shaped into a technique termed X-ray footprinting (XFP, Figure 3B), which is particularly well suited for determining transient conformational states in biomolecules in their native states. Since high-energy X-rays readily penetrate cells and radiolyse water present in the sample on a millisecond timescale, XFP can be deployed as a non-invasive tool to resolve fast structural changes or probe biomolecules *in situ*, even in frozen samples. Focus has remained mostly on studying protein structural dynamics, but the use of XFP for RNA structure elucidation is gaining traction, albeit without any RNP examples yet [47–49]. While XFP relies on highly specialised equipment to produce short-lived ·OH radicals, it addresses important limitations from current methods to probe RNA structure in RNPs and is primed to become a powerful tool to unravel the role of condensates in RNA folding and viral genome packaging.

### Heterogeneity within viroplasms supports particle assembly and virion release from condensates

The existence of distinct assembly stages with few malformed or arrested intermediates suggests that viral particles assemble within viroplasms in a fixed, stepwise manner despite this constant macromolecular flux across membraneless interfaces [16,50]. Though how this occurs is currently unclear, heterogeneity in condensate properties and organisation likely plays a role in this co-ordinated process.

As previously discussed, RNA sequence and structure heavily influence local condensate properties. Super-resolution microscopy of eukaryotic germ granules identified nanoscopic RNA clusters within these condensates that assembled in a sequence-independent manner [51]. Sites of viral particle assembly that concentrate genomic RNA within viroplasms may similarly represent distinct microenvironments analogous to sub-phases, relying on higher affinity binding during assembly to concentrate or exclude condensate components. Colocalisation of rotavirus genomic RNA may also be sequence-independent, instead relying on RNA structure, length, or interactions with proteins before sequence-specific interactions later sort segments for faithful packaging of each segment. For example, the rotavirus non-structural protein NSP2 is abundant throughout viroplasms and has been implicated in the colocalisation of genomic segments through its affinity for non-specific RNA binding. Radiolabelling of particle intermediates has demonstrated that NSP2 is gradually excluded from assembling particles [50]. This may represent an outwards flux of non-structural proteins as higher avidity clients such as decameric inner capsid protein VP2 displace them, forming single-layered particles resembling ‘deflated’ viral particles [16] (Figure 1B). Locally elevated concentrations of structural proteins in these microenvironments would promote rapid and faithful particle assembly.

In this model, colocalisation of RNA and VP2 and co-assembly are expected to generate heterogeneity within viroplasms, with viral subunits concentrated at assembly nucleation sites and depleted from the immediate surroundings. Typically, energetic traps at high local concentrations force subunits to assemble in malformed structures before they can rearrange into the correct geometries [52]. LLPS likely allows viroplasms to avoid these traps by providing a fluid environment that buffers the local subunit concentrations at which functional particle assembly is permitted [20]. Though elevated protein concentrations are predicted to promote aggregation, subunit sequestration in the microenvironments of assembling particles may prevent this [20]. Equally, the nucleation events of particle assembly may deplete the surroundings of viral components. The selective partitioning of viral components into condensates would provide a constant flux from the cytoplasm that supplies assembly reactions and buffers any reductions in local subunit concentrations caused by such nucleation events [20].

Interestingly, the mammalian nucleolus provides models for studying viroplasms, as it is one of the most studied examples of multiphasic condensates. These phase-separating bodies consist of fibrillar cores surrounded by a dense fibrillar component and an outer granular component (GC), together harbouring hundreds of different proteins for diverse regulatory roles, including ribosomal assembly [5]. Similarly to viroplasms, nucleoli rely on directional fluxes to export assembling pre-ribosomal structures in the face of bidirectional diffusion across boundaries [53]. Protein binding simultaneously reduces the multivalency of rRNA, making partitioning into the GC less favourable for maturing ribosomes and increasing the free energy of transfer out of the GC [54]. Recent studies of the compositionally distinct nucleolar sub-phases indicate that differences in local properties further promote bidirectional transport, with differences in charge and pH setting up a proton motive force resembling those seen in biological membranes [55]. Examples of similarly sophisticated inner-condensate organisation in viruses include the biphasic morphology of Orthopneumovirus condensates and genomic RNA clustering in +ssRNA RSV inclusion bodies [56,57]. There is currently no evidence of sub-phases within rotavirus viroplasm condensates, which are likely more uniform scaffold-rich bodies with clients residing closer to the cytoplasmic interface. Nevertheless, partial control of the macromolecular diffusion across interfaces and heterogeneity in organisation may also play important roles in the stepwise assembly and export of RNP complexes at viroplasm surfaces.

Uneven protein distribution within viroplasms may be achieved without the interfacial boundaries present in nucleoli through the propensity of higher-valency scaffolds to exclude low-valency clients from densely packed condensate cores [54]. The less densely packed, client-rich surface region in viroplasms may represent a more fluid environment comparable to the nucleolus outer GC layer, which displays more liquid-like properties than the inner gel-like phases [58]. This fluidity would enable faster colocalisation of viral subunits, making particle assembly most efficient at the interface between phases [52].

Cryoelectron tomography of rotavirus viroplasms supports this theory, as assembling particles are only visible near the periphery of otherwise uniformly dense condensates [16] (Figure 1A). These viroplasms are primarily formed of non-structural scaffold protein NSP5 and client NSP2, along with viral RNA. Structural clients, including coat proteins VP2 and VP6, seemingly cannot infiltrate this dense core to compete for available binding sites and instead appear to reside closer to the surface. In support of this, 3D super-resolution microscopy revealed VP2 and VP6 layers localise concentrically around NSP2 and NSP5, with VP4- and VP7-specific antibodies staining the outermost surrounding layers [59]. It should be noted that though the high-resolution imaging of structural biology studies can provide valuable information on nanoscale structures within condensates, direct visualisation of frozen cells often struggles to identify the assembly sequence of intermediate stages or distinguish them from dead-end pathways. Similarly to rotaviruses, adenovirus genome replication and capsid assembly have been proposed to occur primarily at the periphery of viroplasms [60]. However, preliminary evidence suggests that viral assembly may occur elsewhere in the context of other viral condensates. For example, Avian Orthoreovirus virions appear to assemble and reside deeper within replication factories without relying on surface properties [32]. Interestingly, recent evidence suggests that Mammalian Orthoreovirus (MRV) replication factories vary in their composition and perform distinct functions by supporting genome packaging and replication in ‘core-only’ factories at the cellular periphery, as well as containing fully assembled virions in perinuclear factories [61]. These factories may exhibit distinct surface properties, thus preferentially interacting with either hydrophobic lipid droplets or residing in more hydrophilic microenvironments. Similarly, microtubular networks may provide another interaction interface for some viral condensates. For instance, MRV strain TIL produces filamentous viral factories dependent on microtubular networks, whereas strain T3D replicates in globular network-independent inclusions [62]. As such, condensate interfaces likely affect viral replication even without being sites of viral assembly through their selectivity, ability to remodel nucleic acids, and potentially by aiding protein folding.

Similarly to micelles spontaneously arranging amphiphilic subunits, condensates often minimise interfacial tension and entropy by protecting hydrophobic constituents in their core and preferentially exposing more hydrophilic regions to cytosolic solvent [63,64]. For example, nucleolar subcompartments are arranged into immiscible layers, with more hydrophilic subcompartments always on the outermost layers. Inversion of subcompartment micropolarity reverses layering accordingly [65]. Similar condensate properties may be conserved in viroplasms, which may also become increasingly hydrophilic with radial distance from the hydrophobic scaffold-rich core (Figure 1A). Accordingly, clients with greater hydrophobicity and valency involved in earlier assembly steps would partition differentially within viroplasms to form local environments matching their polarity. Unlike nucleoli, which assemble condensates around pre-rRNA transcripts, viroplasms recruit RNA into existing phase-separating bodies primarily formed of viral proteins NSP5 and NSP2. *In vitro* partitioning assays demonstrated that oligo-peptide condensates favour partitioning of shorter, more flexible ssRNAs into dense cores known to promote duplex unwinding, while stiffer dsRNA molecules of equal length resided closer to the periphery in less dense subphases [22,66]. Heterogeneity in viroplasm properties may similarly enable bidirectional transport of RNA into different regions of the same condensate to promote restructuring, colocalisation, and packaging for subsequent export of DLPs (Figure 1B). Viroplasm surfaces may therefore support sequential and vectorial assembly along a gradient of recruited clients following contra-flow RNA recruitment, lacking the more definitive interfaces of subphases seen in the nucleolus.

## Conclusions and future perspectives

In summary, viruses can form condensates with unique biophysical properties imparted by phase separation that perform the functions necessary for generating infectious particles. Experimental evidence has revealed condensates to demonstrate selectivity that would reduce heterogeneity in the condensate [15,22]. In theory, these properties likely organise the viral assembly components and promote RNA remodelling to maximise productive RNA interactions with the assembling protein capsid subunits. Questions remain over the details underlying the assembly of accurately packaged particles and the contribution of structural changes within viroplasms to these processes. Advancements in methodologies studying RNA structure at fast timescales and in chemically compatible ways can significantly enhance our molecular understanding of RNA-mediated LLPS and provide useful insight into its physiological importance in viroplasms. Due to the universal nature of the physical laws underlying LLPS, findings in tractable systems such as viruses can be extrapolated to other cellular systems and vice versa. An improved understanding of the mechanisms through which condensates support RNA selectivity, restructuring, and co-ordinated assembly in replication factories can therefore benefit studies of other MLOs.

## Perspectives

- Virus-induced biomolecular condensates are ubiquitous, yet their contributions to the spatiotemporal organisation of viral replication and assembly remain obscure.
- The tractability of viral systems due to their lower complexity, combined with the ease of genetic manipulations, makes viruses ideal models for studying the roles of phase separation in biology.
- New tools for probing RNA and protein dynamics in droplets, combined with theoretical simulations, will help explain the complex architecture of viroplasms that support the production of nascent virions in cells.

## Open Access

Open access for this article was enabled by the participation of University of Cambridge in an all-inclusive *Read & Publish* agreement with Portland Press and the Biochemical Society under a transformative agreement with JISC.

## Abbreviations

DLP: double-layered particles
DMSO: dimethylsulfoxide
ER: endoplasmic reticulum
GC: granular component
HRF: hydroxyl radical footprinting
LLPS: liquid-liquid phase separation
MLO: membraneless organelle
MRV: Mammalian Orthoreovirus
RNP: ribonucleoprotein
TLP: triple-layered particle
XFP: X-ray footprinting
